# Graphene-Based Nanocomposites: Synthesis, Mechanical Properties, and Characterizations

**DOI:** 10.3390/polym13172869

**Published:** 2021-08-26

**Authors:** Ahmed Ibrahim, Anna Klopocinska, Kristine Horvat, Zeinab Abdel Hamid

**Affiliations:** 1 Department of Mechanical Engineering, Farmingdale State College, Farmingdale, NY 11735, USA; klopaa@farmingdale.edu; 2 Department of Chemical Engineering, University of New Haven, West Haven, CT 06516, USA; KHorvat@newhaven.edu; 3 Central Metallurgical Research and Development Institute (CMRDI), Cairo 11421, Egypt; forzeinab@yahoo.com

**Keywords:** graphene, graphene nanocomposites, graphene characterizations, polymer nanocomposites, mechanical properties of graphene nanocomposites

## Abstract

Graphene-based nanocomposites possess excellent mechanical, electrical, thermal, optical, and chemical properties. These materials have potential applications in high-performance transistors, biomedical systems, sensors, and solar cells. This paper presents a critical review of the recent developments in graphene-based nanocomposite research, exploring synthesis methods, characterizations, mechanical properties, and thermal properties. Emphasis is placed on characterization techniques and mechanical properties with detailed examples from recent literature. The importance of characterization techniques including Raman spectroscopy, X-ray diffraction (XRD), atomic force microscopy (AFM), scanning electron microscopy (SEM), and high-resolution transmission electron microscopy (HRTEM) for the characterization of graphene flakes and their composites were thoroughly discussed. Finally, the effect of graphene even at very low loadings on the mechanical properties of the composite matrix was extensively reviewed.

## 1. Introduction

This paper provides a critical review of the synthesis, properties and characterizations perspectives of recent advances in graphene-based nanocomposites. Section 2 presents an overview of the importance of the graphene properties and prospect applications in smart phones, ultra-thin flexible displays, hydrogen storage, transparent touchscreens, chemical sensors, biosensors, and super-fast transistors. Section 3 and Section 4 summarize the most frequent graphene synthesis techniques including mechanical exfoliation, liquid-phase exfoliation and chemical synthesis technique. They also highlight the polymer nanocomposite processing methods and the morphological states for graphene-based polymer nanocomposites.

A critical review of the characterization of graphene and graphene nanocomposites was presented in Section 5. Detailed research results of graphene characterization from recent literature are thoroughly discussed. Different types of microscopic and spectroscopic characterization methods to obtain structural and morphological data are presented. Mechanical properties of graphene-based nanocomposites are thoroughly discussed in Section 6. In addition, a summary from recent research that exemplifies the effect of graphene’s filler on the improvement of mechanical properties of graphene-based polymer nanocomposites is also thoroughly discussed. Two major tables summarizing the reinforcing effect of graphene-based materials on mechanical properties and thermal conductivity have been constructed. The thermal properties of graphene and graphite nanocomposites are subsequently discussed in Section 7. The variation in thermal conductivity with different forms of graphene and graphite nanocomposites from recent research are summarized.

## 2. Graphene

**Figure 1:**
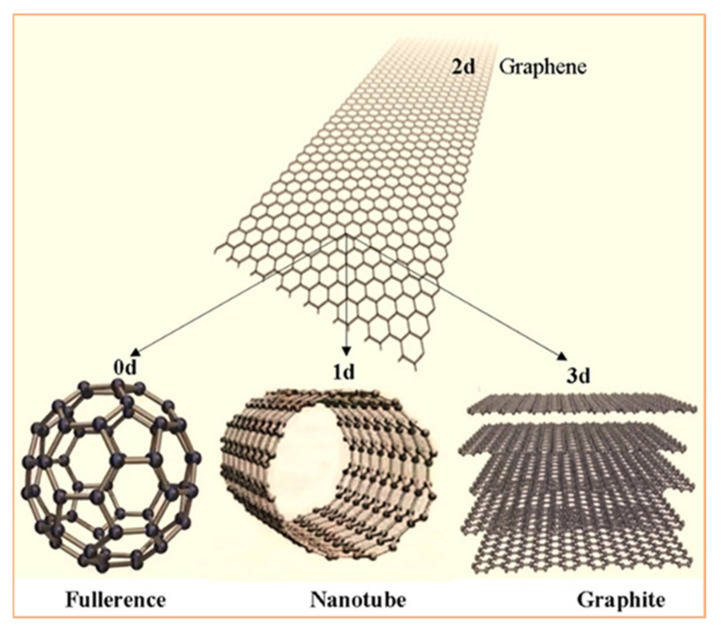
Crystal structures of different allotropes of carbon: graphite (3D); graphene (2D); nanotubes (1D); and fullerene (buckyballs) (0D).

Carbon has several allotropes, which can be classified according to the type of chemical bond related with hybridization (sp, sp^2^, sp^3^): zero-dimensional sp^2^ fullerenes, the two-dimensional sp^2^ honeycomb lattice of graphene, or three-dimensional sp^3^ crystals—diamond [1,2,3,4]. Each allotrope has different electronic and mechanical properties. Graphene, fullerenes, and carbon nanotubes (CNTs) are emerging new materials with superior properties (Figure 1). The great versatility of carbon materials arises from the strong dependence of their physical properties on the ratio of sp^2^ ~graphitelike to sp^3^ ~diamondlike bonds [4]. There are many forms of sp^2^-bonded carbons with various degrees of graphitic ordering, ranging from microcrystalline graphite to glassy carbon. Accordingly, these materials have been greatly investigated because of the exceptional mechanical and electronic properties.

Graphene is an allotrope of carbon consisting of a single layer of atoms arranged in a two-dimensional honeycomb lattice [1]. Carbon atoms are bonded with a covalent sp^2^ bond with a single free electron, which accounts for the conductivity of graphene. Graphene is attracting great interests from the physical, chemical, and biomedical fields as a novel nanomaterial with exceptional physical properties, including extremely high thermal conductivity, excellent electrical conductivity [1,2,3,4,5], high surface-to-volume ratio, remarkable mechanical strength, and biocompatibility [6,7,8,9,10,11]. Graphene possesses unique electronic properties and is recognized as the most thermally conductive known material [12,13,14,15,16,17]. Experimental results show that graphene has a remarkably high electron mobility at room temperature [12,18], and has been considered as an alternative in transistor circuitry. The electron mobility in graphene is almost 200 times higher than Si and 4 times larger than III–V semiconductors [15]. This would make graphene a very attractive material for high-speed transistors.

Since its discovery in 2004, graphene has become the center of many research activities [1,9,19,20,21,22,23,24,25,26,27,28,29,30]. It is a unique type of carbon where every atom is accessible for chemical reaction because of its 2D structure. With a Young’s modulus (stiffness) of 1 TPa, it is the strongest material ever tested [8]. Graphene possesses other remarkable characteristics: electron mobility is 100× faster than silicon; its electrical conductivity is 13× better than copper; it conducts heat 2× better than diamond; and it has a high surface area of about 2630 m^2^/gram. Over the past decade research on graphene increased dramatically because of new methods to produce and study it. Graphene and functionalized graphene (FG) have been successfully used in many applications including in smart phones, ultra-thin flexible displays [31], hydrogen storage [32], transparent touch-screens [33], chemical sensors effective at detecting explosives [34,35], biosensors, super-fast transistors [36,37,38], and so on. Graphene has been investigated for tissue engineering [39]. It has also been utilized as a reinforcing agent to enhance the mechanical properties of biodegradable polymeric nanocomposites for bone tissue applications.

Graphene reveals remarkable optical properties, which makes it very promising for photonic and optoelectronic applications [31,40,41]. It is nearly transparent to visible light as well as to UV and IR. Graphene can be used to conduct electricity away from the solar panel as part of a light and flexible solar panel. However, the proportion of the defects in the structure of graphene has a great influence on the physical and mechanical properties. Graphene nanocomposites (GNP) possess a high aspect ratio, which makes them ideal for reinforcement [42,43,44,45,46,47,48,49]. The set of remarkable properties of graphene-based systems has expanded into new fields of investigation. Graphene is truly a multi-disciplinary material, being researched in many different fields for various potential applications. The optical of graphene represents potential fields of significant research and application.

## 3. Methods of Graphene Preparation

Graphene has great prospects for industrial applications, such as polymer composites, conductive coatings, fuel cells batteries, and ultracapacitors due to its distinctive properties of high strength and exemplary electrical and thermal conductivity [2,3]. These applications demand large quantities of graphene in the form of nanoparticles or nanoplatelets at a reasonable price. Several approaches have been used to prepare graphene. Mass production of high-quality graphene (single or few layers graphene) is a major challenge. Structural disorders, defects, and wrinkles within the graphene may have a detrimental impact on its electronic properties [50].

**Figure 2:**
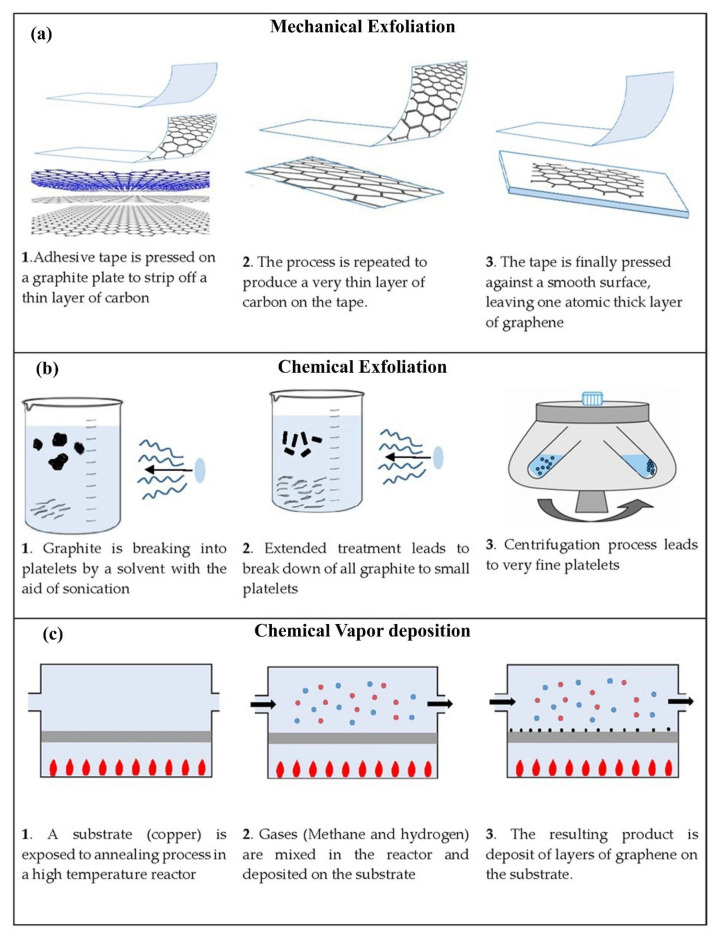
A schematic showing the main graphene production techniques. (**a**) Mechanical exfoliation. (**b**) Chemical exfoliation. (**c**) Chemical vapor deposition.

There are several approaches available to produce graphene (Figure 2). These techniques are: mechanical exfoliation, liquid-phase exfoliation, and chemical vapor deposition (CVD). These techniques could be grouped into two categories, i.e., bottom-up (CVD) and top-down (exfoliation methods) processes. Each technique has distinct advantages as well as limitations depending on its intended application. A brief description of these techniques follows.

### 3.1. Exfoliation

Most of the graphene produced for research over the past decade was fabricated by mechanical exfoliation (scotch tape method) [51,52,53]. In this method, samples of graphite are placed on the sticky area of an adhesive tape pressed on a desired substrate and then peeled away (Figure 2a). Flakes of graphene (only a few microns wide) are left on the substrate [9]. The graphene developed by the scotch tape process is of very high quality and enables researchers to measure its physical and mechanical properties. However, this method is not practical for producing graphene on a large scale for industrial applications. To be effective for a solar cell application, for example, graphene must cover the entire surface of the cell, not just a partial area.

### 3.2. Liquid-Phase Exfoliation

Liquid-phase exfoliation (LPE) (Figure 2b) is one of the most feasible production methods because of its scalability for commercial manufacturing of graphene at low cost. This method is very versatile and applicable to different environments and on various substrate types. Liquid-phase exfoliation of graphite requires wet chemical dispersion then sonication in appropriate solvents [54]. It involves three different steps: (1) dispersion in a solvent, (2) exfoliation, and (3) purification in order to separate the exfoliated material. The mechanism of exfoliation is attributed to the force induced by ultrasound and the interaction with the solvent molecules. The principle of LPE lies in assisting the separation between graphene layers. In graphite, they are held together by strong electrostatic attractions that require a large amount of mechanical force to separate. One way to reduce this energy input is to disrupt the attractive forces holding layers of graphene together. This is achieved by first immersing the bulk material in a special liquid, followed by exfoliation. Nuvoli et al. obtained a high concentration of few-layer graphene sheets by liquid-phase exfoliation of graphite in ionic liquid [55]. Phase exfoliation of graphene from bulk graphite is a versatile top-down approach for producing high-quality graphene samples. LPE techniques have several advantages, including a relatively low cost for high yield and the ease of scaling up. High-quality graphene production methods are crucial for harnessing graphene’s properties for future applications as a material with various applications [55].

### 3.3. Chemical Vapor Deposition (CVD)

The CVD method is widely used for the synthesis of carbon nanostructures (CNTs) for composite materials with outstanding mechanical properties [56,57,58]. Figure 2c shows how graphene can be created by thermocatalytic decomposition of gaseous hydrocarbons onto a metal surface. CVD is a relatively new technique for producing films of large area of continuous, 2D graphene. During the CVD process, a metal substrate such as copper is placed into a furnace and heated under low vacuum. Gases (methane and hydrogen) run through the furnace. The hydrogen then catalyzes a response between the methane and the area of the metal substrate, producing carbon atoms in the methane to be settled onto the outer lining of the metal. The resulting product is a deposit of layers of graphene on the substrate [59]. Copper is not the only substrate that may be utilized in graphene CVD—quite few other transition metals can be used as well. For example, graphene CVD on nickel and cobalt have also been performed. [60]. The CVD technique allows for precise control of the number of layers grown.

## 4. Graphene-Based Nanocomposites

Research on polymer nanocomposites (PNC) has been growing over the past decade due to their remarkable material properties, yield strength, toughness, electrical conductivity, thermal conductivity, and optical properties, and their applications are growing substantially [2,61,62,63,64,65,66,67,68,69,70,71,72,73,74,75,76,77,78,79,80]. Traditional composite structures contain a significant amount (~50 vol%) of filler bound in a polymer matrix—PNC typically—containing a small amount of inorganic particles (usually 1 to 3 wt%) and size less than 100 nm, with a very large surface area dispersed in the polymer matrix [79]. However, it has been shown that a graphene of micron-size could be made scalable to mass production [81]. This makes graphene-based composite materials appealing to a great number of applications [81].

Graphene possesses many desirable properties such as high strength and elastic modulus, high electrical and thermal conductivity, high aspect ratio, high thermal stability, high gas impermeability, and good dimensional stability [6,7,8,9,10,11,12]. Polymer properties can be dramatically improved by the addition of graphene at a low volume fraction. Moreover, graphene has a higher surface-to-volume ratio than CNT and can be used at a lower volume fraction than CNT. It is potentially more promising for improving many properties of polymer matrices.

Graphene can be produced in large quantities from graphite precursor by oxidation. Hence, graphene-based polymer nanocomposites have attracted considerable research interest around the globe. Various polymers, such as epoxy [82,83,84,85,86,87,88], PMMA [89,90,91,92,93,94,95], HDPE [96], polystyrene [96,97,98,99,100,101], and nylon [91,102,103,104,105,106,107] have been used as matrices to fabricate graphene polymer nanocomposites. Malucelli et al. (2016) provide an excellent summary of the preparation of graphene-based nanocomposites [82]. It is worth noting that the quality of graphene dispersion in the polymer matrix directly correlates to its effectiveness in improving the nanocomposites’ properties [108,109]. The properties of a composite are also closely related to the aspect ratio of the graphene filler.

Graphene-based nanocomposites are increasingly being used for the development of new materials for alternative energy sources, for example, (a) in lithium-ion batteries, graphene-based nanocomposites show better performance as they have high power density and energy density and a fast charging speed in hydrogen fuel cells; (b) graphene is used as an electrode material to enhance electrocatalytic activity; (c) in solar cells, graphene-based composites are used in photovoltaic devices because of their unique characteristics of high carrier mobility and low resistivity; and (d) in thermoelectric materials. 

Characterization of nanocomposites is crucial to understand the basic physical and chemical properties of the nanocomposites. Graphene materials available in the market are made by different companies through different techniques. It is expected that these graphenes are quite different from each other in flake width, thickness, and defect concentration. All the different techniques to modify the filler surface as well as to synthesize the polymer nanocomposites need to be supplemented with robust characterization of these processes as well as resulting composite properties to gain insight into the various factors affecting the nanocomposite microstructure. Several techniques have been used in the characterization of nanocomposites, dispersion, distribution, and orientation within polymer matrix. These techniques include optical microscopy, scanning electron microscopy, high transmission electron microscopy, Raman spectroscopy, atomic force microscopy and X-ray diffraction; these have been shown to be very useful for quantification of nanocomposites. It is also, in many instances, necessary to employ more than one characterization technique in order to accurately characterize the nanocomposite material.

### 4.1. Polymer Nanocomposite Synthesis

Nanocomposites contain matrices of diverse materials such as polymer, metal or ceramic, and also include different nanoparticle fillers (graphene, nanotubes, clays). These fillers enhance the mechanical, thermal and electrical properties of the material [110,111,112]. The polymeric type nanocomposites are by far the most versatile and their application is widespread in many diverse industrial fields such as energy, electronics, biomedical, etc.

**Figure 3:**
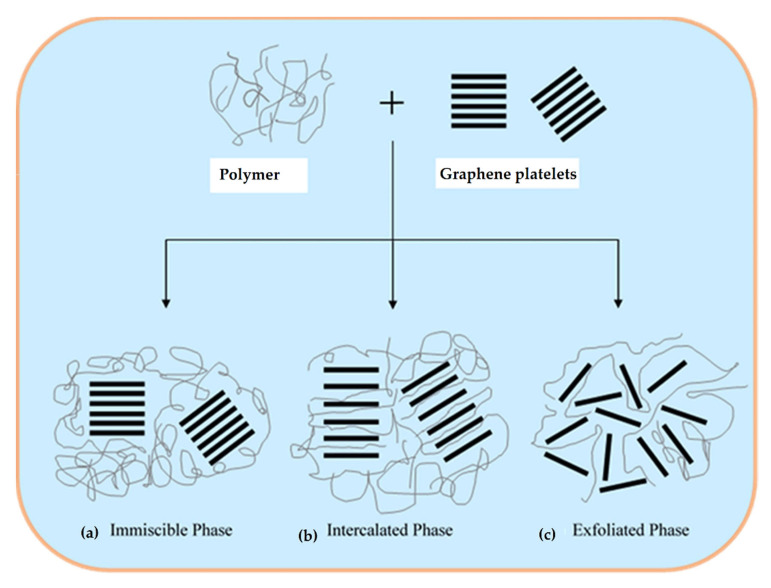
Schematic showing three morphological states for graphene-based polymer nanocomposites. (**a**) Immiscible phase. (**b**) Intercalated phase. (**c**) Exfoliated phase.

Depending upon the degree of dispersion of the nano-sized layer structure, polymer composites can be divided into three main categories: microcomposites, intercalated nanocomposites, and exfoliated nanocomposites [109,113,114]. In the microcomposites’ structure (Figure 3a), graphene sheets are dispersed inside the polymer matrix in the form of particles and the graphene platelets remain intact. When individual polymer chains are introduced between graphene layers, intercalated constructions are obtained (Figure 3b). In the exfoliated hybrids (Figure 3c), graphene layers are homogenously dispersed in the polymer matrix. The exfoliation configuration is the preferred morphology for nanocomposites as it maximizes the area of contact between the polymer and the filler and results in stronger bonding, and remarkable mechanical properties [108].

**Figure 4:**
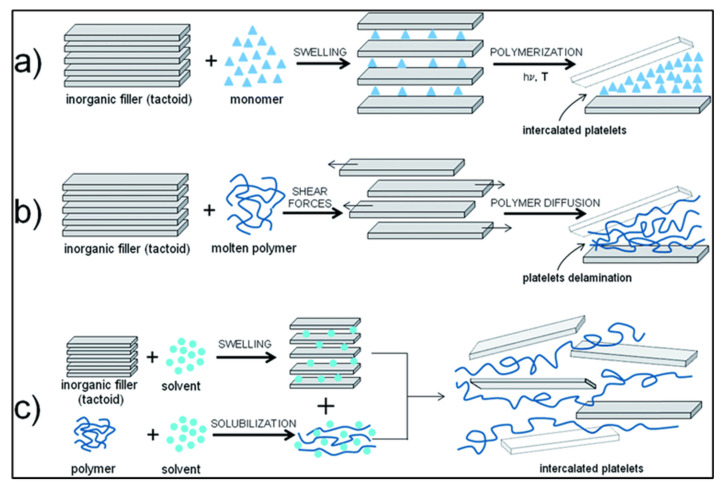
Synthesis of nanocomposites (**a**) in situ polymerization, (**b**) melt processing, and (**c**) exfoliation adsorption. Adopted from [114] (CC BY).

The properties of graphene nanocomposites are dependent on chemical compatibility between the filler and the matrix, the volume fraction of the filler and the processing conditions such as dispersion and exfoliation of filler. To achieve optimal results, appropriate fabrication methods must be employed [59,60]. Moreover, the performance quality of nanocomposites is strongly associated with the degree of dispersion [107,114,115,116,117,118]. Methods of polymer nanocomposite synthesis can be divided into three main categories: in situ polymerization, melt intercalation, and exfoliation adsorption (Figure 4).

#### 4.1.1. In Situ Polymerization

In situ polymerization is an effective technique for the formulation of highly dispersed graphene in a polymer matrix. This process involves the polymerization of monomer in the presence of the layered materials (Figure 4a). During in situ polymerization, the filler is placed into a monomer solution. The filler swells, allowing the monomer to seep in between its layers. The solution is stirred and sonicated. When even distribution is achieved, polymerization is initiated using heat, radiation, initiator diffusion, or by the addition of catalyst. As polymer chains are created in between and around layers of the filler, they force the layers apart, leading to improved exfoliation. In situ polymerization provides excellent dispersion and greater compatibility between graphene and the polymer through the introduction of added functional groups. Unlike other synthesis methods, this process allows for the use of thermoset polymers [114]. The key advantage of this technique is the potential for formation of strong covalent bonds between the nanofiller and matrix during polymerization [113,114].

#### 4.1.2. Melt Blending

Melt blending is the preferred method for the synthesis of various polymer hybrids with inorganic nanoparticles [15]. It is found to be cost-effective and environmentally friendly. One of the main advantages of this method is that it does not require any type of solvent, and the graphene or treated graphene can be directly mixed in the molten polymer matrix. This method is the conventional method for the mixing of the thermoplastic polymer with the graphene or modified graphene. Examples of such methods are extrusion and injection molding. The main disadvantage of this method is the poor dispersion of the graphene in the polymer matrix, specifically in higher filler loadings. This happens because of the increased viscosity of composites.

The most common method of synthesizing polymer nanocomposites is melt intercalation or melt blending [109,117,119]. Melt blending encompasses melting of polymer to create a viscous liquid. The nanofillers are dispersed into the polymer matrix using a high shear mixer along with a high temperature [119,120,121]. In this process, shown in Figure 4b, the polymer matrix is heated to its glass transition temperature, the filler is added, and the mixture is kneaded until uniform distribution is achieved. The degree of intercalation is dependent on processing conditions such as mixing speeds and times, temperature, compatibility of the polymer matrix and the fillers, the filler’s interlayer forces, and filler surface preparation [105]. Melt intercalation is free from solvents and can be incorporated into plastic manufacturing processes such as injection molding and extrusion, making it convenient and economical. Fillers with a high degree of thermal stability are required to prevent filler degradation due to high temperatures required for manufacturing and processing [105]. Bao et al. [122] produced graphene/polylactic acid (PLA) nanocomposites with outstanding properties. The PLA nanocomposites demonstrated well-dispersed graphene and significant improvement in crystallinity, mechanical properties, and electrical conductivity. The major drawbacks to this method are increased viscosity at high filler fractions, and a lesser degree of intercalation than with other methods [112]. Although melt blending is a convenient method for generating composites with enhanced quality, it is less effective in dispersing nanofillers compared to solution blending.

#### 4.1.3. Exfoliation Adsorption

A more effective way to achieve interfacial adhesion between the filler and matrix is exfoliation adsorption (Figure 4c) [114,122,123,124]. Exfoliation adsorption, also called polymer intercalation from solution, or solution mixing, requires solvent compatibility between the filler and matrix [113]. Both the filler and matrix are dissolved then mixed together. The solvent causes the filler to swell, increasing interlayer space and allowing the polymer chains to intercalate in between the layers of the filler. The mixture is stirred and sonicated to obtain an even distribution and the solvent is removed by evaporation or precipitation. After solvent removal, polymer chains become entrapped between layers of the filler, forming a multilayer structure. This process is used for creating nanocomposites from polymers with low polarity but is not ideal for industrial use due to the large quantities of solvent required [104]. The solution mixing can be used to obtain polymer nanocomposites with a range of polymers, such as poly(methylmethacrylate) (PMMA) [125], polyurethane (PU) [126], and poly(vinyl alcohol) (PVA) [49].

## 5. Characterization of Graphene and Graphene Nanoplatelets (XGnPs)

Graphene is a material with outstanding properties, such as high specific surface area 2600 m^2^/g, high mobility (15000 cm^2^/V·s) [10,15], superior thermal conductivity (3000 W/m·K) [127], and extremely low permeability. Topological defects exist in large-area polycrystalline graphene [127,128,129,130], and are thought to play crucial roles in tailoring mechanical and physical properties of graphene [127,131,132,133,134]. Thus, characterization of graphene is an important step for understanding graphene’s properties. The overall electronic properties and the purity of a graphene sample are determined by the number of layers present. Characterizations of graphene encompass different types of microscopic and spectroscopic methods to obtain structural and morphological data of the synthesized graphene. Similarly, the characterization process is also related to determining of the purity and defects of graphene. Synthesis processes and/or processing parameters have a great effect on graphene’s purity. HRTEM and AFM are commonly used to determine number of layers of graphene. On the other hand, Raman spectroscopy is commonly employed to characterize the purity of graphene and to measure the number of its layers by detecting various crystal structures and bonding information. Furthermore, XPS and Raman spectroscopy are the fundamental methods for the measurement of graphene’s chemical purity and detection of functional groups attached to the graphene.

### 5.1. Raman Spectroscopy

Raman spectroscopy is a standard nondestructive tool for the characterization of crystalline, nanocrystalline, and amorphous carbons [135,136,137,138,139,140,141,142,143,144,145,146,147,148,149,150,151]. It is a high-resolution tool for the characterization of the lattice structure and the electronic, optical, and phonon properties of carbon materials, including three-dimensional (3d) diamond and graphite, 2d graphene, 1d carbon nanotubes, and 0d fullerenes. Raman spectroscopy is a powerful and reliable tool for the characterization of graphene family materials due to its sensitivity to the vibration of C–C bonds [135,136,137]. It is a highly sensitive method to determine and quantify the density of defects in graphene [135]. As the process of making graphene is very diverse, including mechanical exfoliation, chemical vapor deposition, and chemical exfoliation, several types of carbon can exist as byproducts. Raman is a very powerful technique that can be of great benefit for characterization of carbon nanomaterials.

**Figure 5:**
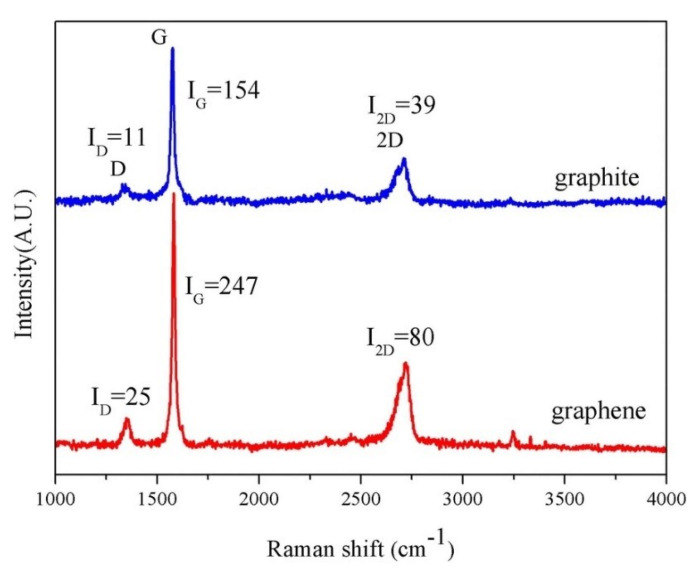
Raman spectroscopy of the bulk graphite and graphene [140] (CC BY).

Zhiliang et al. [140] demonstrated a simple method based on hydrodynamic mechanisms for production of high-quality graphene flakes. A simple needle valve was used as an exfoliation device. The results indicated that ~71% of the prepared graphene flakes were less than five layers, while the average thickness and length of the flakes were 2.3 nm. Figure 5 shows a typical Raman spectrum of the prepared graphene along with the bulk graphite as a reference. Three typical characteristic peaks, i.e., D band (~1350 cm^−1^), G band (~1580 cm^−1^), and 2D band (~2700 cm^−1^), were observed for these two graphitic materials. The intensity ratio of D/G (*I*_D_/*I*_G_) for the prepared graphene was 0.10, lower than that of ultrasonication exfoliated graphene (0.29) [152].

**Figure 6:**
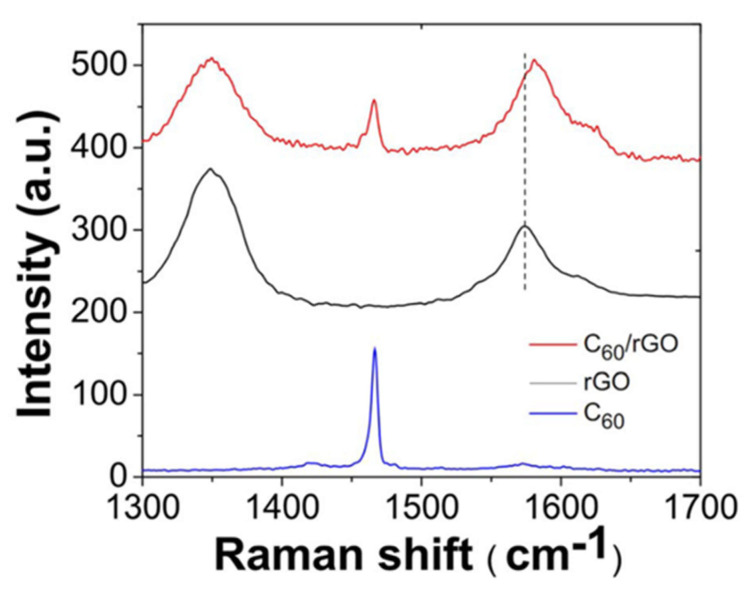
Raman spectra of C60, rGO and C60/rGO hybrid. Adopted from [152] (CC BY).

Zhang et al. [152] carried out an investigation to enhance the thermoelectric properties of organic composites. Functionalized graphene was combined with a semiconductive fullerene, and then the fullerene-coated graphene was integrated into a conjugated polymer. Graphene helps enhance electrical conductivity, while fullerene hinders thermal conductivity, resulting in a synergistic effect to enhance thermoelectric properties. Electrical conductivity increased by seven-fold and thermal conductivity increased by ten-fold. The reduced graphene oxide (rGO) and C_60_/rGO samples were characterized by Raman spectra. Figure 6 shows Raman spectra of the graphene, pristine C_60_, and C_60_ graphene. The two intense peaks in rGO are designated to the D and G band. The G band and D band are a result of the presence of defects that have been introduced throughout the oxidization and reduction technique. The G band has moved to 1582 cm^−1^ in the C60/graphene hybrid, indicating the effect of graphene on C_60_.

**Figure 7:**
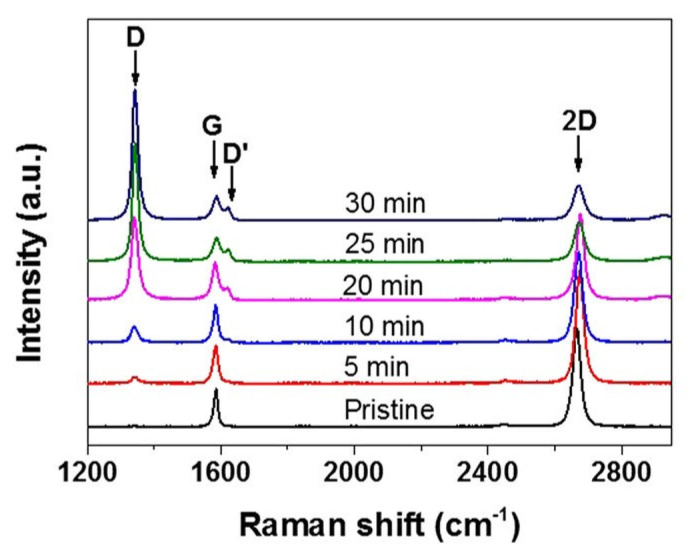
Raman spectra of pristine graphene and BPO-functionalized graphene heated at 80 °C for 5 min, 10 min, 20 min, 25 min and 30 min in nitrogen atmosphere, respectively. Adopted from [159] (CC BY).

Chemical functionalization of graphene (CFG) enables graphene to be processed by solvent assisted techniques, such as layer-by-layer assembly, spin-coating, and filtration. It also prevents the agglomeration of single-layer graphene during reduction and maintains the inherent properties of graphene [153,154,155,156,157,158]. CFG is of great importance for many applications include electronics and conductive graphene films for touch screen. Gao et al. [159] developed a heat-initiated chemical reaction to functionalize CVD-grown graphene. Figure 7 shows the time evolution of the Raman spectra for functionalized graphene heated at 80 °C in nitrogen atmosphere. As the reaction time increased, the characteristic disorder-induced D band (1330 cm^−1^) emerged as the most important feature of the Raman spectra. In addition, the double 2D band considerably weakened, while the G band around was broadened due to the presence of a defect.

### 5.2. Atomic Force Microscopy (AFM)

Atomic force microscopy (AFM) is one of the most powerful microscopy techniques for studying samples at nanoscale. AFM is a type of scanning probe microscopy (SPM), with a resolution on the order of fractions of a nanometer [160]. For example, AFM provides 3D images of the graphene film, its thickness, and the number of layers present. Modern AFM imaging provides the most consistent, highest resolution AFM imaging. Hence, an AFM can be employed to verify the thickness of graphene films. Graphene fillers usually exhibit various morphologies (e.g., folded, crumpled, and distorted sheets). AFM, similar to the SEM and TEM methods, can also be used for studying the shape, size, structure, absorption/dispersion, and aggregation of nanomaterials. There are several scanning modes employed in AFM studies, i.e., static mode (noncontact mode), contact mode, dynamic mode, and tapping mode.

**Figure 8:**
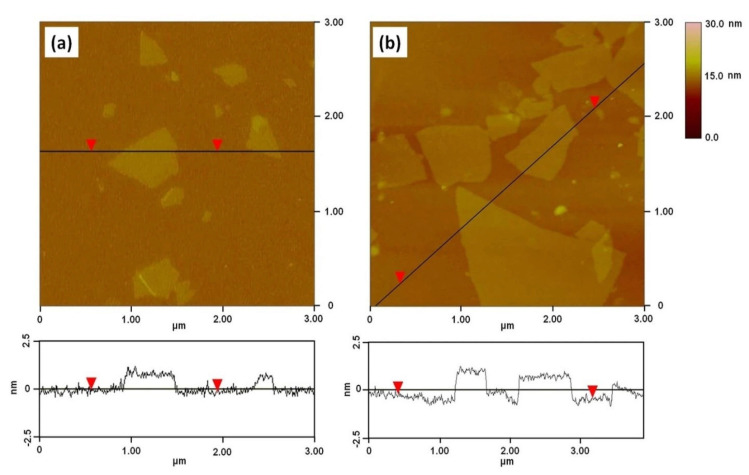
Atomic force microscopy (AFM) images and cross-sectional analysis of (**a**) GO and (**b**) S-rGO [161] (CC BY).

Reduced graphene oxides (rGO) are single-layered sheets derived from the chemical reduction of graphene oxide (GO). The rGO possess electrical and mechanical properties similar to those of graphene; thus, this makes them versatile for a number of applications. Various synthetic routes have been reported for the synthesis of reduced graphene oxide [142,161,162,163,164,165,166,167,168]. Lu et al., developed a facile method to synthesize sulfonic acid-grafted graphene oxide, S-rGO, as an effective catalyst to prepare Pt/S-rGO electrocatalysts via a self-assembly route [161]. The morphologies of the GO and S-rGO were examined in detail by AFM. The AFM images confirm that the GO and S-rGO are comprised of isolated and well dispersed GO sheets, as shown in Figure 8. Moreover, The GO sheets have lateral dimensions of several micrometers and a thickness of 0.8 nm, which is characteristic of a fully exfoliated GO sheet.

**Figure 9:**
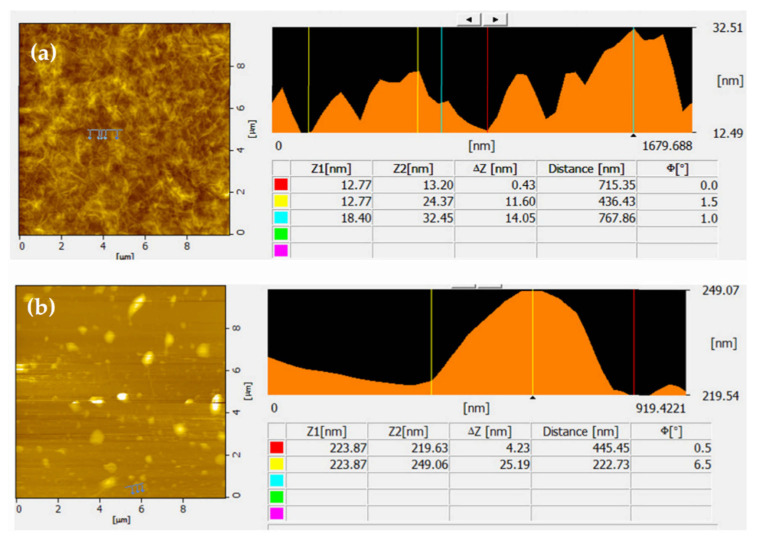
Characterization atomic force microscopy images of (**a**) graphene oxide and (**b**) bacterially reduced graphene oxide [163] (CC BY).

**Figure 10:**
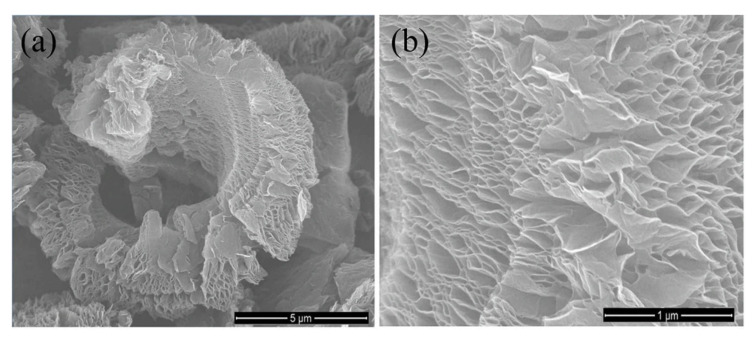
SEM images of reduced graphene oxide (rGO). SEM images at (**a**) low and (**b**) high magnification [162] (CC BY).

Gurunathan et al., demonstrated the synthesis of water-soluble graphene through reduction of GO using bacterial biomass [163]. The proposed approach confers that bacterially reduced graphene oxide (B-rGO) has great potential for various biological and biomedical applications. AFM images were used to characterize the surface morphology and thickness of the GO and B-rGO nanosheets. Figure 9 shows images of graphene oxide (A) and bacterially reduced graphene oxide (B). These images clearly indicate that GO exhibits flat sheets and an average thickness of about 0.43 nm, indicating the formation of single-layered GO nanosheets. On the other hand, the B-rGO was thicker at ~4.23 nm, demonstrating that the biomass adhered and reduced the GO surface successfully (Figure 10b).

### 5.3. Scanning Electron Microscopy (SEM)

Graphene fabricated by different techniques contains many defects depends on the process. Defects in graphene have negative impacts on the high mobility and other physical and mechanical properties of graphene. Accordingly, it is essential to characterize these defects using SEM or other advanced microscopy techniques. SEM is an advanced microscopy instrument in characterization of micro- and nanostructured materials. Along with its high power to offer a 2D analysis of substantial aspects of the sample, SEM can also provide information and varied qualitative data on several physical properties such as roughness, morphology, surface consistency, size, and chemical structure of materials. Nano scale features of graphene including wrinkles, grain shapes, and folding lines can be specifically characterized utilizing the SEM [161,162].

Figure 10 shows high-quality SEM images of graphene nanosheets produced for future application as a cathode material for sodium-ion batteries [162]. In this study, graphene oxide was synthesized by a modified Hummers’ method and reduced using a solid-state microwave irradiation method. The SEM images revealed a wrinkled stack of ultra-thin graphene oxide nanosheet with a porous morphology. A high-magnification SEM image (Figure 10b) shows a large number of nanopores between the nanosheets that are formed by gas evolution. Nanoporous carbon materials have attracted considerable technological interest due to their numerous applications, including improving the tensile strength of composites, as catalyst and sensor supports, as hydrogen-storage materials, and in electronic and electrochemical devices [1].

**Figure 11:**
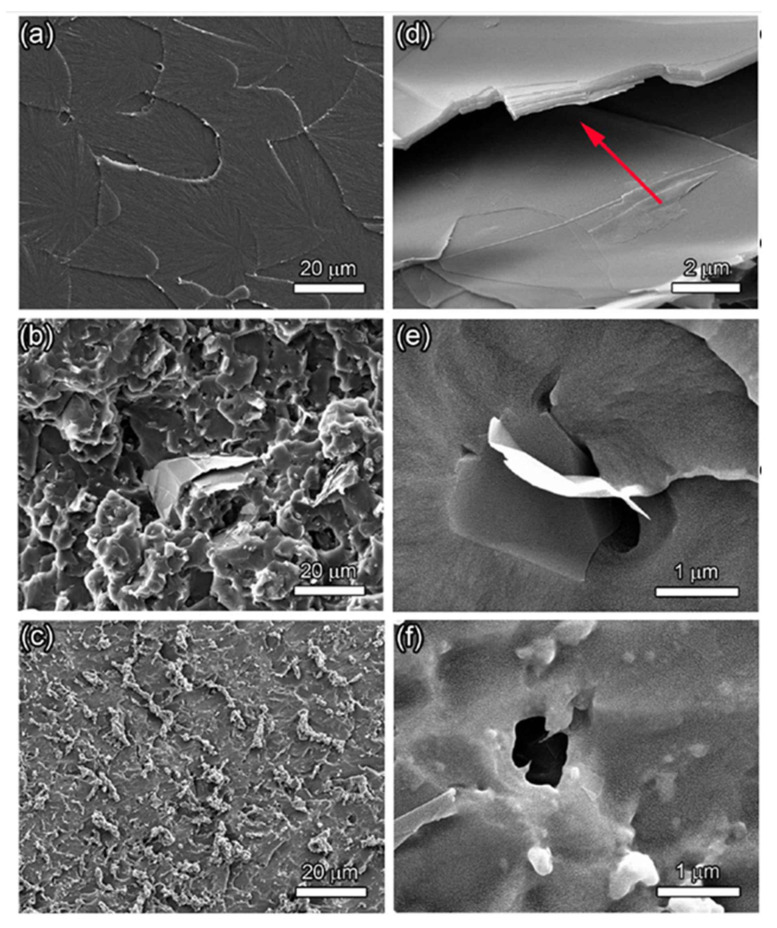
Mechanical characterization and morphology of 1.0 wt.% graphene–epoxy composites. (**a**–**c**) SEM images of fracture surfaces of epoxy resin, ultrasonic treated nanocomposites, and the (HP and ultrasonic) treated nanocomposites, respectively. (**d**) High magnification image of graphene block in (**b**) The big gap between the graphene sheets, as indicated by the arrow in (d), implies that the graphene sheets slide over each other during the bending test. (**e**,**f**) High magnification images of wrinkled and bridging graphene in (**c**). Reproduced with permission from Nature Publishing group [164] (CC BY).

Many studies demonstrated that the state of dispersion of graphene sheets in the matrix has a strong effect on the mechanical properties of the composite [161,164,165,166,167,168,169]. Yang et al. [164] demonstrated that graphene strengthening could be improved by the excellent dispersion from the hydrogen passivation (HP) and ultrasonication technique. Figure 11 shows the SEM images of the fracture surfaces of a pure epoxy, graphene composites produced by ultrasonication, and by the HP and ultrasonication [161]. The fracture surface of pure epoxy is relatively smooth (Figure 11a). Compared to the fracture surface of the pure epoxy, the fracture surfaces of graphene/epoxy (Figure 11b) composites are rough and consist of many small facets, an indication that the graphene inhibits fracture of the composites and thus results in a rougher fracture surface. The graphene sheets dispersed by the HP and ultrasonication technique (Figure 11c) attained much better dispersion in the epoxy matrix compared to the graphene reinforced composites fabricated by ultrasonication alone (Figure 11b). Figure 11d shows a high magnification image of graphene layers (Figure 11b) that were separated during the three-point bending test, which may have resulted from a weak adhesion between layers. Such morphology usually has a negative effect on the mechanical strength of composites. In contrast, the graphene dispersed by the HP and ultrasonication was thickly coated with an adsorbed epoxy layer (Figure 11e). In addition, graphene linking was often observed on the fracture surface (Figure 11f), indicating strong epoxy–graphene interaction.

**Figure 12:**
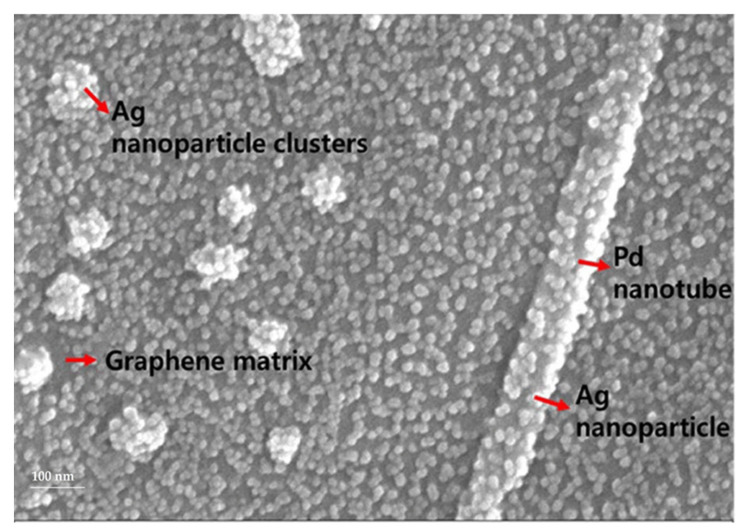
SEM image of graphene–Pd–Ag on sensing FETs [165] (CC BY).

Graphene is quite a robust material in hydrogen sensors due to a possible improvement of its surface area and susceptibility of its electronic properties to the changes caused by adsorbing atoms and molecules including hydrogen. However, the pristine graphene sensitiveness to hydrogen is limited [34]. Sharma et al., developed a dual FET hydrogen gas sensor using graphene decorated Pd-Ag alloy nanoparticles for H_2_ detection [165]. Figure 12 shows the SEM image of the graphene–Ag–Pd nanocomposites on the sensing area of the sensor platform. The integration between graphene, Pd and Ag can be visualized from the SEM image. Ag nanoparticles with the size of Ca. 17 nm and Pd nanoparticles at the size of Ca. 100 nm are uniformly and compactly embedded on the graphene layer. The morphology and nature of the Pd–Ag films grown on the graphene substrate are clearly shown in Figure 12.

### 5.4. High Resolution Transmission Electron Microscopy (HRTEM)

HRTEM is a very powerful structure characterization technique for graphene. It is a unique tool for characterizing the atomic structures and interfaces of graphene [170,171]. It has been exploited to reveal the fine chemical structure of graphene oxide and observe graphene flakes in a fraction of micron. It is a very high-resolution TEM with advanced imaging features that are superior to conventional TEM. HRTEM can be employed in graphene-based catalyst characterization to determine the number of graphene layers, which directly determines the surface area of the catalyst and further affects their catalytic performance dramatically [171]. HRTEM can also be used to estimate thickness of a graphene sample. Accordingly, the number of layers can be calculated. The other direct method is the observation of the edges by HRTEM, which provides an accurate way to count the number of layers at multiple locations on the layer-structure catalyst. HRTEM elemental mapping is also a powerful tool to detect the element distribution of graphene-based catalysts.

Due to strong interactions and van der Waals forces, exfoliated graphene sheets have a strong tendency to irreversibly aggregate or even restack, reverting to multilayer structures such as graphite [172]. Therefore, functionalization must be performed to reduce hydrophobicity, and to increase dispersion in organic and aqueous solutions. Covalent functionalization is the addition of molecules to graphene that results in the rehybridization of the sp^2^ carbon atoms of the π network into a sp^3^ configuration, resulting in adjustments of the innate physical and chemical properties of graphene [172].

**Figure 13:**
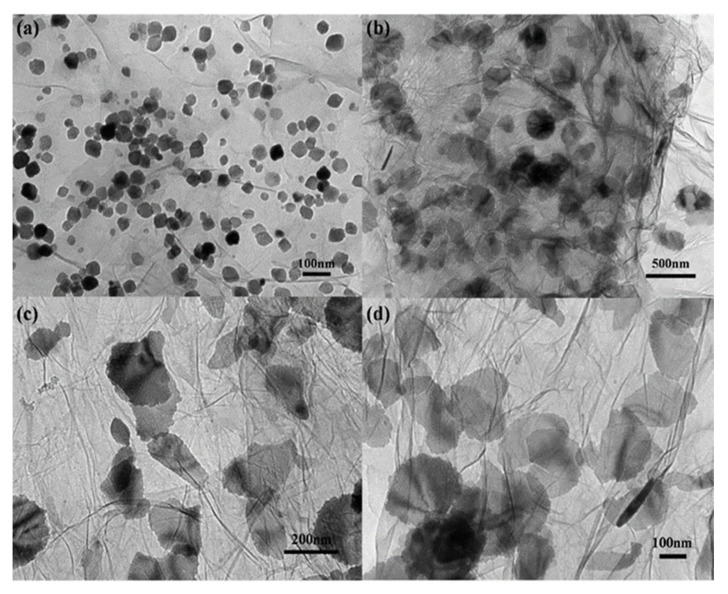
The morphological analysis of Fe_2_O_3_–graphene nanocomposites by TEM. TEM images of (**a**) Fe_2_O_3_–graphene particle-on-sheet composite, and (**b**–**d**) Fe_2_O_3_–graphene sheet-on-sheet composite with stepwise increased magnifications [173] (CC BY).

The lithium-ion battery is considered to be one of the best power sources to maximize the efficiency of energy use. Graphite has been widely used as an anode material for commercial Li-ion batteries because of its good electrochemical properties. Kan et al. [173] prepared a new Fe_2_O_3_–graphene structure, namely sheet-on-sheet nanostructure by a solvothermal method. Fe_2_O_3_ nanoparticles were also prepared on graphene nanosheets. The Fe_2_O_3_–graphene sheet-on-sheet nanostructure was fabricated as an anode for Li-ion batteries. It was confirmed that Fe_2_O_3_ nanosheets were uniformly dispersed among graphene nanosheets, forming a unique sheet-on-sheet nanostructure. HRTEM of the morphological analysis of Fe_2_O_3_–graphene nanocomposites is shown in Figure 13. A large number of pristine Fe_2_O_3_ nanoparticles are shown in this figure. Their particle sizes are 30–50 nm size. The particle-on-sheet and sheet-on-sheet nanostructures can be easily confirmed by the HRTEM images of Figure 13a–d, respectively. These nanoparticles and nanosheets are clearly uniformly distributed on graphene nanosheets.

**Figure 14:**
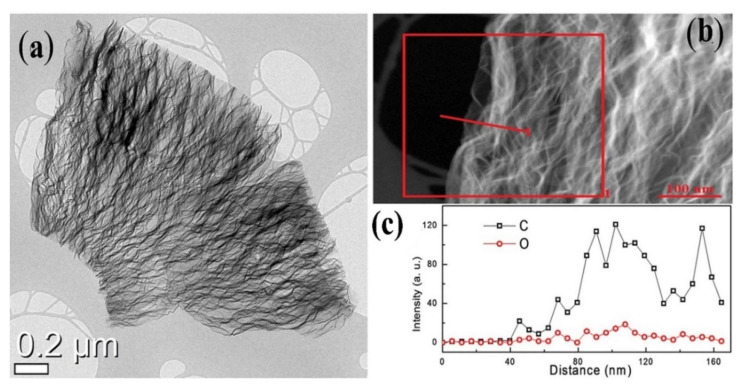
(**a**) HRTEM images of the RGO stack. (**b**) EDS line-profile showing the functional groups on RGO, and (**c**) relative signals of C and O from the EDS line-profile image [162] (CC BY).

Figure 14 [162] shows a HRTEM image of a reduced graphene oxide (RGO) produced for future application as a cathode material for sodium-ion batteries. The RGO morphology reveals an interesting doughnut-like morphology. Figure 14b,c show that the EDS profile clearly demonstrates the existence of a functional group on the RGO. The relative contents of C and O were detected and plotted against distance (Figure 14c). The EDS results indicated that the RGO was greatly functionalized to perform more effectively for charge storage.

### 5.5. X-ray Diffraction (XRD)

XRD is an important analytical tool for the characterization of the intercalated and exfoliated nanocomposites [172,173,174,175,176]. XRD can measure accurately the interlayer or basal plane d-spacing, for example, of GO and monitor intercalation of any species in the gallery of the GO lattice. Whereas the interlayer spacing of graphite is 3.35 Å, conversion to GO results in an increase in this basal plane spacing due to functionalization of graphite with oxygen-containing groups. X-ray diffraction (XRD) has been used widely in the structural characterization of sp^2^ carbon materials [176]. XRD patterns of graphite and graphene have distinct peaks and can be used to differentiate between graphite and graphene structures. For example, the intense peak at 2θ = 26.3° graphite shifts to 14.1°–14.9° in graphite oxide. However, XRD peaks disappear as the sheets of GO exfoliate into single sheets [149,150].

The application of X-ray diffraction (XRD) for phase determination and confirming the reduction of GO to graphene is a very useful tool. Of course, it is not as powerful as the other approaches (Raman spectroscopy, FTIR, AFM) due to the limited information collected. Therefore, its application is always accompanied by other methods such as Raman, XPS, and FTIR to provide more information about the catalyst structure.

**Figure 15:**
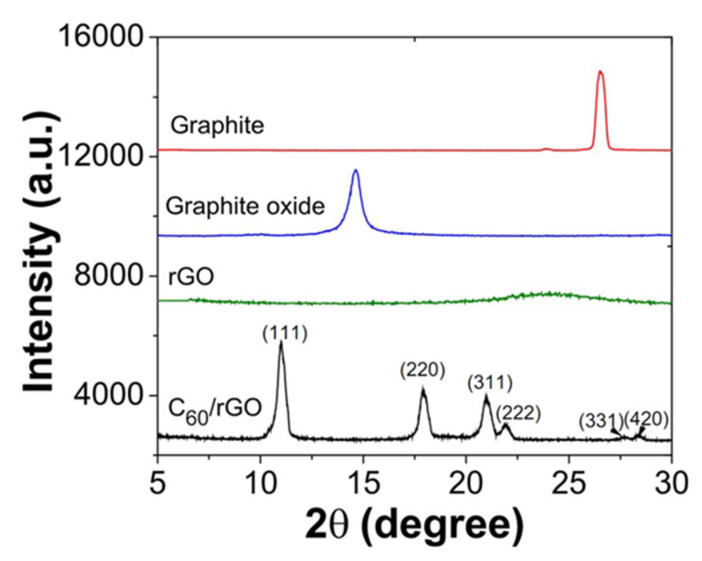
XRD diffraction patterns of graphite and its derivatives [152] (CC BY).

Polymer-based thermoelectric materials have been the focus of many studies for the past 10 years [177,178,179,180,181]. Zhang et al. [152] prepared thermoelectric polymer composite by functionalized graphene with fullerene (C60), and then dispersed fullerene in the polymer. The XRD of graphite and C60/rGO are shown in Figure 15. The XRD pattern of graphite shows a characteristic peak at 2θ = 27°. After oxidation, the graphitic peak shifts to 14.6°, demonstrating that the interlayer spacing increased. After chemical reduction, the graphite oxide peak disappeared. It was suggested that the sharp peak is attributed to the exfoliation of layered structures of graphite oxide [182]. The XRD pattern also clearly indicates that fullerenes (C_60_) had been effectively integrated into the surface of graphene.

**Figure 16:**
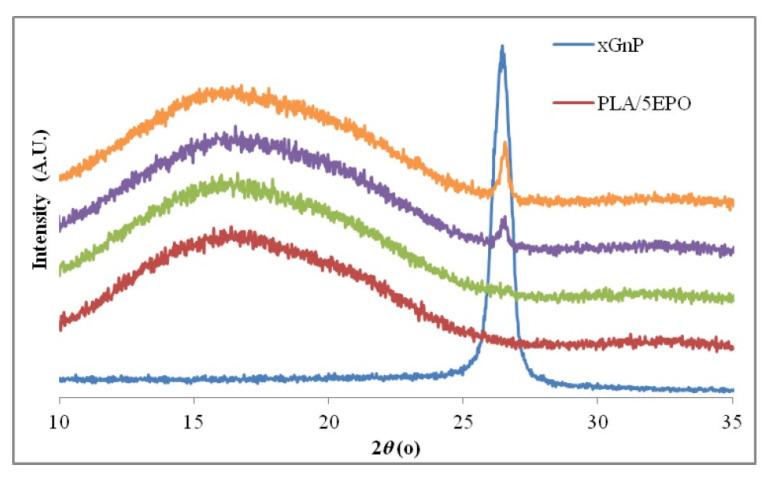
XRD patterns of different graphene nanocomposites. Reproduced from [183] (CC BY).

Chieng et al. [183] prepared nanocomposite by blending poly(lactic acid) (PLA)/epoxidized palm oil (EPO) with graphene nanoplatelet (xGnP). The PLA/EPO/xGnP green nanocomposites were characterized by XRD. Figure 16 displays the XRD spectra of xGnP, PLA/EPO and PLA/EPO composite with different xGnP loading. The xGnP displays a strong peak at 2θ = 26.4°. The XRD spectra of PLA/5EPO blend and PLA/5EPO with different xGnP loadings display a broad characteristic peak of PLA matrix at 2θ = ~16°. PLA/5EPO nanocomposites with 0.3 wt.% xGnP loading display a small peak around 26.5°, which corresponds to the characteristic peak of xGnP.

## 6. Mechanical Properties of Graphene-Based Nanocomposites

Graphene-based nanocomposite materials exhibit significant improvement in mechanical properties compared to the standard composites [2,46,184,185,186,187]. Graphene has been commonly used to strengthen mechanical properties of several nanocomposites [185,186]. For example, the tensile strength of the baseline epoxy was enhanced by ~40% with graphene platelets with addition of a very small weight % of graphene (~0.1 wt.%) [46]. The inclusion of nanoparticles within the matrix enhances linking of polymer chains (crosslinking density) of the nanocomposites and resulted in a remarkable improvement in the mechanical properties. These properties are mainly correlated to the number of graphene layers and the interior defects of the graphene structure. For example, monolayer graphene sheets exhibit excellent mechanical properties (E ~ 1 TPa and strength ~ 130 GPa) [8]. 

Table 1 demonstrates the percentage enhancement in the mechanical properties, elastic modulus, and tensile strength of graphene polymer nanocomposites compared to the polymer matrix [187,188,189,190,191,192,193,194,195,196,197,198,199,200,201,202,203]. Table 1 is constructed based on the type of graphene (GNP, GO, rGO) and then categorized based on the polymer matrix that has been used in each case. Mechanical properties of the nanocomposites have been investigated by several techniques including nanoindentations [204,205,206,207,208].

Most of the properties of the nanocomposites were superior compared to the polymer matrix [209,210,211]. This is often attributed to the graphene filler’s very high aspect ratio. In a comprehensive study [4], it was demonstrated that the addition of 1.0 wt.% of graphene to PMMA leads to an increase of 80% in the elastic modulus and 20% in increase in ultimate tensile strength. The same study reported that the single-layer functionalized graphene (FGS) gives the best results compared to other nanofillers (SWNT, EG). 

Pinto et al. [187] investigated the effect of incorporating graphene oxide and graphene nanoplatelets on mechanical properties of poly(lactic acid) (PLA) films. The results indicated that incorporation of very small loadings of GO or GNP (0.2 to 0.6 wt.%) in PLA films significantly improves mechanical properties. This confirms the reported potential of graphene-related materials in providing relevant performance gains at low loadings, because of the high specific area available for interaction with the polymer matrix. An optimum loading was identified for mechanical performance, corresponding to about 0.4 wt.% for both materials. 

King et al. [193] fabricated epoxy nanocomposites by incorporating 1–6 wt.% GNP in the epoxy. These composites were tested for tensile properties using typical macroscopic measurements. Nanoindentation was also used to determine modulus and creep compliance. These macroscopic results showed that the tensile modulus increased from 2.72 GPa for the neat epoxy to 3.36 GPa for 6 wt.% GNP in epoxy composite. The modulus results from nanoindentation followed this same trend.

Li et al. [197] prepared polyvinyl alcohol (PVA) composite fibers reinforced with graphene reduced from graphene oxide (GO). After reduction, most of the oxygen-containing groups were removed from the GO and reduced graphene oxide (rGO) was prepared. The PVA/rGO composite fibers exhibited a significant enhancement of mechanical properties at low rGO loadings; in particular the tensile strength and Young’s modulus of the 2.0 wt.% rGO and PVA composite fiber increased to 244% and 294%, respectively, relative to neat PVA fibers. 

**Figure 17:**
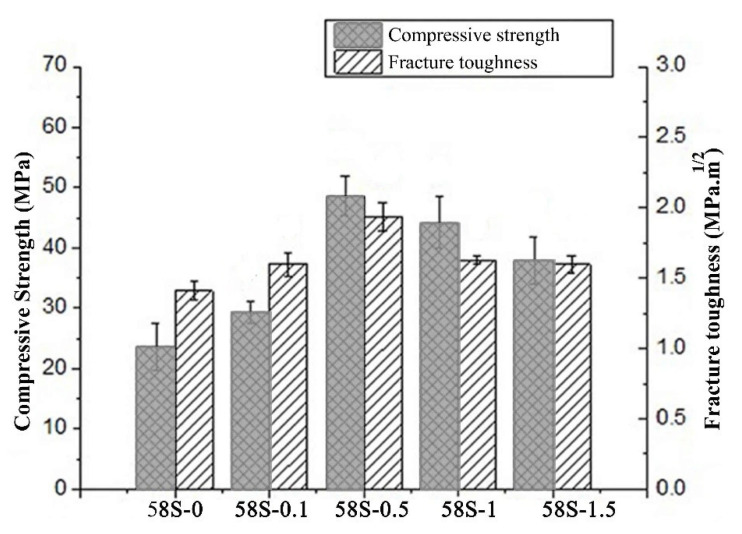
Mechanical properties of graphene nanocomposites. Reproduced from [212] (CC BY).

**Figure 18:**
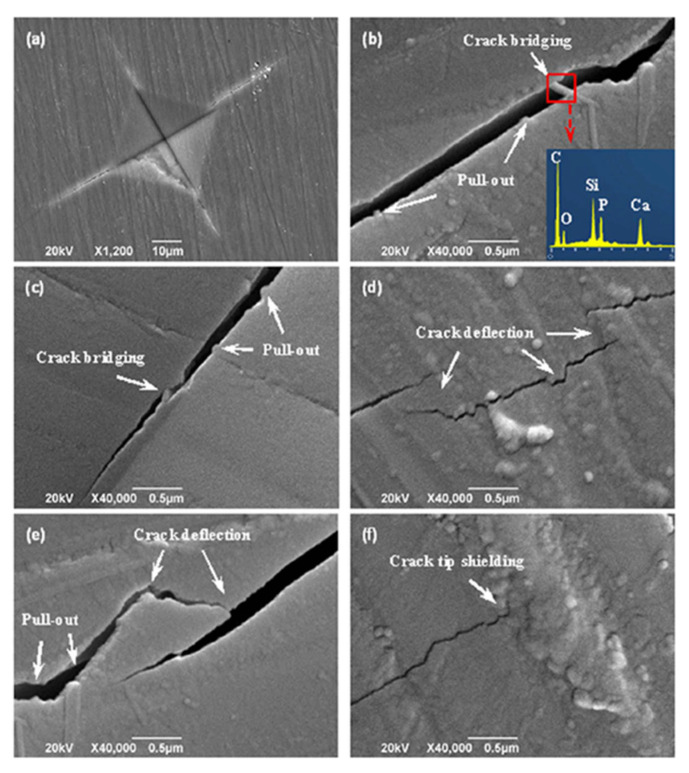
Crack propagation by graphene in the sintered samples. SEM images of (**a**) microhardness in dentation, (**b**–**e**) crack deflection, crack bridging and graphene pull-out, (**f**) closure of crack growth at the crack tip. Reproduced from [212] (CC BY).

Gao et al. [212] combined Nano-58S bioactive glass with graphene to enhance its mechanical and biological performance for bone tissue engineering applications. Figure 17 shows the compressive strength and fracture toughness of nanocomposite scaffolds of graphene/nano-58S. Analysis of the data in Figure 17 shows that the fracture toughness was ~1.95 MPa ∙ m^1/2^ with a graphene loading of 0.5 wt.%, suggesting major improvements due to graphene. The microhardness indentations and radial cracks on the polished surface of 58S-0.5 were characterized by SEM to identify the mechanism responsible for the enhanced mechanical properties (Figure 18). Figure 18b shows the presence of graphene crack bridging on the fracture line. EDS analysis shows a strong peak of carbon element confirming the toughening effect of graphene. Figure 18b–e) show the mechanism of crack bridging in graphene/nano-58S. Based on this investigation, it was concluded that graphene was extremely effective in slowing crack propagation in the matrix.

**Figure 19:**
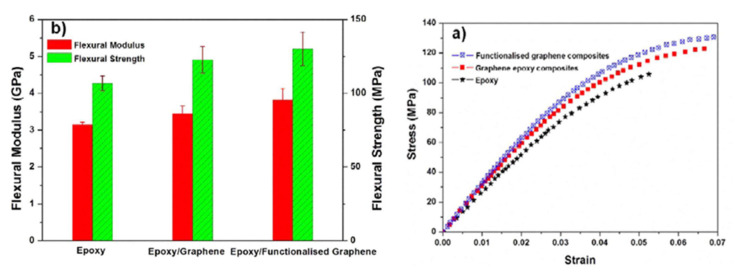
(**a**) Stress–strain diagrams; (**b**) bending modulus and strength of the composite samples. Reproduced from [213] (CC BY).

Thermally reduced graphene oxide (TRG) can be produced via the rapid heating of GO under inert gas and high temperature (1000 °C) [213]. TRG is a top-down method for bulk production of graphene. TRG sheets contained from a single layer to a few layers of graphene with the average size of 500 nm [214]. Outstanding properties of graphene make it an excellent filler for polymer nanocomposite applications. Naebe et al. [214] mixed TRG with epoxy resin after being functionalized to produce epoxy nanocomposites. The mechanical properties and morphology of the nanocomposites were investigated to assess the effect of the functionalization on the dispersion in the produced composites. The modulus of elasticity and the flexural stress–strain were determined by the three-point bending test. The test was carried out to investigate the effect of addition of FG on the mechanical properties of the epoxy matrix. Figure 19 shows stress–strain diagram for the composites of TRG/FG. The addition of TRG and FG results in an increase in the flexural strength of the epoxy matrix by 15% and 22%, respectively.

Nanoindentation is a widely used technique for measuring the nano and microscale mechanical properties in nanomaterials and nanocomposites. This technique has been commonly used in determining the mechanical properties of polymers [204,205,206,207,208]. Recently, it has been used to measure mechanical properties of nanocomposites. Shokrieh et al. [206] used nanoindentation to evaluate the mechanical properties of graphene nanocomposites. According to this investigation, the mechanical properties of a pure polymer matrix are remarkably improved by the addition of small amounts (0.05 wt.%) of graphene nanoplatelets (GNP).

Shen et al. [207] used nanoindentation to study mechanical properties of clay nanocomposites with different polymer matrices. Aldousiri et al. [208] used nanoindentation to measure the modulus and the hardness of polyamide-layered silicate nanocomposites. Lee et al. [8] evaluated the elastic properties and fracture strength of monolayer graphene by nanoindentation. The force displacement behavior yields elastic stiffness, E, of 340 N m^−1^. The fracture strength that signifies the intrinsic strength (σ_int_) of the sheet was ~42 N m^−1^. The corresponding values for the bulk graphite were E = 1.0 TPa and σ_int_ = 130 GPa.

## 7. Thermal Properties of Graphene and Graphene Nanoplatelets (xGnPs)

All electronic units produce excessive heat and thus demand thermal management to prevent premature failure [215,216,217,218,219,220]. Thermal management is crucial for the efficiency of the advanced integrated circuits (ICs) and high-frequency high-power density communication devices. Recently, use of high-conductivity materials is suggested for electronic cooling and for improving the heat dissipated from chips. The cost of high conductivity materials is of major concern. Therefore, there is a real need for low cost high thermal conductivity materials and efficient design to integrate these materials in electronic devices.

Graphene has drawn tremendous attention for heat dissipation due to its extraordinarily high in-plane thermal conductivity (2000~4000 Wm^−1^K^−1^) compared to copper (400 Wm^−1^K^−1^). The thermal properties of graphene have become an important research topic and are attracting tremendous interest in the area of thermoelectric waste heat recovery. Thermal properties of graphene are related to its low mass and the strong bond of carbon atoms. Thermal properties of graphene and its use in electronics and thermal management applications have been the subject of many scientific studies [220,221,222,223,224,225,226,227,228,229].

**Figure 20:**
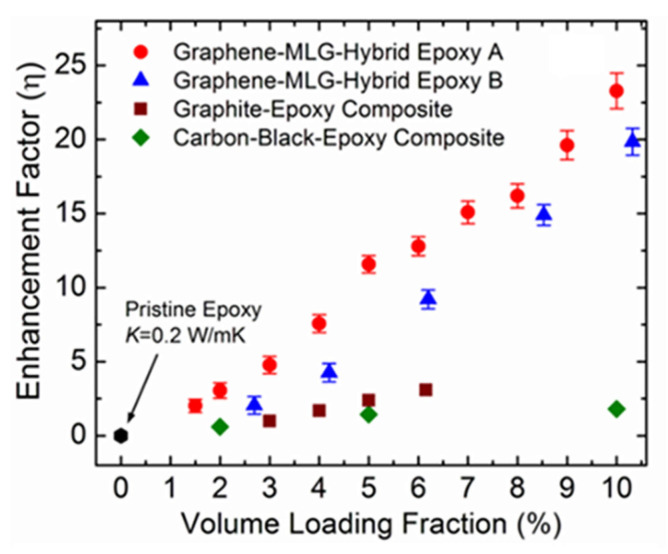
Thermal conductivity enrichment in the graphene–epoxy nanocomposite. (**a**) Thermal conductivity enrichment factor as a fraction of the filler volume loading fraction [220]. Reproduced with permission from the American Chemical Society (ACS).

Shahil et al. [220] synthesized graphene−MLG nanocomposite polymer TIMs and demonstrated the extremely high TCE factors at low filler loadings. They demonstrated a great improvement in thermal conductivity of thermal interface materials (TIMs). They achieved that by optimizing a mixture of graphene and multilayer graphene (MLG). The thermal conductivity of the epoxy matrix material was increased by a factor of 23 at the 10% volume of graphene loading (Figure 20). Moreover, the epoxy–graphene nanocomposite conserved all the properties of TIM needed for industrial applications.

Yu et al. prepared graphite nanoplatelets (GNPs) by thermally exfoliating graphite [222]. Results of thermal conductivity showed that a small quantity of graphene (*n* = 4) was a very effective nanofiller for epoxy composites. They reported a remarkable increase in thermal conductivity (3000%) at 25 vol% loading. They attributed the remarkable increase in thermal properties of this composite to the optimum combination of the high aspect ratio, stiffness, and low thermal interface resistance of the GNPs.

Zhang et al. [226] fabricated a novel composite for a high performance thermal interface system. They arranged graphene sheets vertically in a liquid polydimethylsiloxane (PDMS). They reported a remarkable increase (3329%) in the thermal conductivity of the graphene/PDMS composite. They postulated that this enhancement is due to the vertical alignment of graphene films with high in-plane thermal conductivity, which forms a rapid and effective heat-transfer path.

The variation in thermal conductivity with different forms of graphene and graphite nanocomposites is reviewed in Table 2 [230,231,232,233,234,235,236,237,238,239,240]. The remarkable improvement in thermal conductivity was noted in the case of XGnP compared to other fillers. Graphene-based polymer nanocomposites showed tremendous improvement in electrical conductivity. The remarkable improvement in electrical conductivity resulted from the creation of a conductive network by the graphene in the polymer matrix. In addition to filler type, enhancement in thermal conductivity depends on other factors including the processing method and the polymer matrix. For example, Zhou et al. prepared a composite by adding 2 wt.% multi-layer graphene oxide (MGO) to an epoxy resin, and the thermal conductivity of the composite reached a maximum 2.03 times that of the epoxy [230]. The presence of 2 wt.% MGO percolating chains leads to a sharp rise in the energy barrier. Renteria et al. reported that functionalization of LPE graphene and few-layer-graphene flakes with Fe_3_O_4_ nanoparticles allowed them to align the fillers in an external magnetic field during dispersion of the thermal paste to the connecting surfaces [240]. The filler alignment results in a strong increase in the apparent thermal conductivity and thermal diffusivity.

## 8. Conclusions

This article reviewed the recent progress in graphene-based polymer nanocomposites, focusing on two areas: properties and characterization. Emphasis was placed on the unique roles and advantages of advanced graphene characterization techniques, such as Raman spectroscopy, XRD, AFM, and HRTEM. Characterization of 2D graphene involves different types of microscopic and spectroscopic techniques to obtain the structural, morphological, and chemical information of as-synthesized graphene. Raman spectroscopy is a remarkable analytical tool that can detect small changes in the structural morphology of carbon nanomaterials. It is used to determine the number of layers, defects, strain, and chemical modifications. HRTEM is one of the most powerful characterization techniques for graphene’s structural characterizations. Several examples of graphene polymer composite characterizations using Raman, SEN, TEM, AFM, XRD, and HRTEM were discussed.

Most common graphene synthesis techniques have been discussed. The mechanical exfoliation of graphene is a simple technique for graphene fabrication. The process has the ability to fabricate a single crystal of graphene with various numbers of layers. However, control over the wafer scale synthesis and reproducibility is very difficult. Liquid-phase exfoliation is one of the most practical methods for commercial manufacturing of graphene because of its low cost and potential scalability. Graphene prepared by this technique exhibited a low percentage of defects and oxygen functional groups.

Chemical synthesis techniques are suitable for low volume production of graphene at low temperature. Furthermore, the process can yield graphene film or graphene coatings on several substrate materials. Graphene synthesized using chemical processes exhibits a high surface area and easy functionalization. CVD is widely used for the synthesis of carbon nanostructures. This method has been effectively used for producing carbon filaments which are the basis for composite materials with outstanding mechanical properties.

Graphene–polymer nanocomposites display remarkable mechanical properties compared to the pure polymer. Table 1 shows the percentage enhancement in the mechanical properties, elastic modulus and bending strength of nanocomposites. Most of the properties of nanocomposites were significantly higher than the polymer matrix. This remarkable improvement was related to the filler’s very large aspect ratio.

Graphene and xGnP have attracted tremendous attention for heat removal due to their extraordinarily high in-plane thermal conductivity (2000~4000 Wm^−1^K^−1^). Graphene and MLG, produced by the liquid-phase exfoliation technique, were successfully used to fabricate novel nanocomposites to be used as TIM in electronic applications. Remarkable enhancement of thermal conductivity of the epoxy matrix (increase by 23×) was achieved at the 10% volume of graphene loading. Graphene and xGnP are potential key materials for the next generations of ICs and 3D electronics. Graphene chemical functionalization caused outstanding improvement in the thermal conductivity of the polymer composites. Thermal conductivity is affected by several factors, such as the graphene loading, dispersion, and the thermal resistance of the interface.

## Figures and Tables

**Table 1:** Mechanical properties of graphene polymer nanocomposites.

Graphene	Matrix	Process	Filler Loading (wt.%)	Matrix Modulus (GPa)	Tensile Modulus Increase (%)	Graphene Modulus E (GPa)	Ref
GNP	PLA	Solution blending	0.4 wt.%	0.038	156	250	[187]
GNP	PP	Melt mixing	10 wt.%	1.3	41	13	[188]
GNP	PP	Melt mixing	1.7 vol%	1.3	54	14	[189]
GNP	Epoxy	Solution blending	5 wt.%	2.5	28	30.5	[190]
GNP	Epoxy	Solution blending	1 wt.%	2.9	24	143	[191]
GNP	Epoxy	Solution blending	5 wt.%	2.7	49	55	[192]
GNP	Epoxy	Shear mixing	6 wt.%	2.72	23.5	20	[193]
GNP	Epoxy	Shear mixing	4 wt.%	2.7	8	11	[194]
GNP	PE	Melt mixing	4 wt.%	1.3	35	25	[195]
rGO	PE	polymerization	5.2 wt.%	0.23	170	15	[196]
rGO	PVA	Wet spinning	2 wt.%	5.4	294	1036	[197]
rGO	Epoxy	polymerization	2 wt.%	0.48	70	34	[198]
rGO	Epoxy	Three roll mill	8 wt%	2.8	22	14	[199]
fGr	Epoxy	Solution blending	0.2 wt.%	2.9	8	243	[200]
fGr	Epoxy	Solution blending	0.3 wt%	1.5	32	321	[201]
GO	PVA	Solution blending	0.3 wt.%	2.3	150	2335	[202]
GO	PVA	Solution blending	5 wt.%	2	190	162	[203]

**Table 2:** Thermal conductivity values of graphene polymer nanocomposites.

Matrix	Filler Type	Filler Loading (wt.%)	% Increase in Thermal Conductivity	Reference
Epoxy	MGO	2	104	[230]
Epoxy	GO	1	4.8	[231]
Epoxy	GNP	8	627	[232]
Epoxy	GNP	25	780	[233]
Epoxy	GO	3	90	[234]
Epoxy	GNP	5	240	[235]
Epoxy	GNP	4	700	[236]
Epoxy	GNP	1.9	9	[237]
Epoxy	RGO	1	44	[238]
Epoxy	NG	1	24	[239]
Epoxy	MGO	2	95	[240]

## Data Availability

Not applicable.

## Abbreviations

CVD	Chemical vapor deposition
FGr	Functionalized Reduced Graphene Oxide
GO	Graphene oxide
RGO	Reduced graphene oxide
TRGO	Thermally reduced graphene oxide
GIC	Graphite intercalation compounds
CNT	Carbon nanotube
SWCNT	Single-walled carbon nanotube
MWCNT	Multi-walled carbon nanotube
EG	Expanded graphite
GNP	Graphite nanoplatelets
HDPE	High density polyethylene
PET	Poly(ethylene terephthalate)
PMMA	Poly(methyl methacrylate)
PP	Polypropylene
XGnP	Graphene nanoplatelets
FG	Functionalized graphene
PVA	Poly(vinyl alcohol);EVA, ethylene vinyl acetate
PS	Polystyrene
PU	Polyurethane
PLA	poly(lactic acid)
MGO	Multi-layer graphene oxide
NG	Natural graphite
GF	Graphite flakes
MLG	Multilayer graphene
IM	Thermal interface materials
LPE	Liquid-phase exfoliation
